# Episodes of strain experienced in the operating room: impact of the type of surgery, the profession and the phase of the operation

**DOI:** 10.1186/s12893-020-00937-y

**Published:** 2020-12-07

**Authors:** Sandra Keller, Steven Yule, Douglas S. Smink, Vivian Zagarese, Shawn Safford, Sarah Henrickson Parker

**Affiliations:** 1 Fralin Biomedical Research Institute at Virginia Tech Carilion, Roanoke, VA USA; 2 Center for Surgery and Public Health, Brigham and Women’s Hospital, Boston, MA USA; 3 STRATUS Center for Medical Simulation, Boston, MA USA; 4 Department of Surgery, Brigham and Women’s Hospital, Harvard Medical School, Boston, MA USA; 5 Department of Clinical Surgery, University of Edinburgh, Edinburgh, Scotland UK; 6 Department of Psychology, Virginia Tech, Blacksburg, VA USA; 7 Division of Paediatric Surgery, Virginia Tech Carilion School of Medicine, Roanoke, VA USA; 8 Center for Simulation, Research and Patient Safety, Carilion Clinic, Roanoke, VA USA

**Keywords:** Operating room, Strain, Stress, Tension, Phase, Surgeon, Anesthetist, Scrub technician, Circulating nurse, Student

## Abstract

**Background:**

Strain episodes, defined as phases of higher workload, stress or negative emotions, occur everyday in the operating room (OR). Accurate knowledge of when strain is most intense for the different OR team members is imperative for developing appropriate interventions. The primary goal of the study was to investigate temporal patterns of strain across surgical phases for different professionals working in the OR, for different types of operations.

**Methods:**

We developed a guided recall method to assess the experience of strain from the perspective of operating room (OR) team members. The guided recall was completed by surgeons, residents, anesthesiologists, circulating nurses and scrub technicians immediately after 113 operations, performed in 5 departments of one hospital in North America. We also conducted interviews with 16 surgeons on strain moments during their specific operation types. Strain experiences were related to surgical phases and compared across different operation types separately for each profession in the OR.

**Results:**

We analyzed 693 guided recalls. General linear modeling (GLM) showed that strain varied across the phases of the operations (defined as before incision, first third, middle third and last third) [quadratic (F = 47.85, p < 0.001) and cubic (F = 8.94, p = 0.003) effects]. Phases of operations varied across professional groups [linear (F = 4.14, p = 0.001) and quadratic (F = 14.28, p < 0.001) effects] and surgery types [only cubic effects (F = 4.92, p = 0.001)]. Overall strain was similar across surgery types (F = 1.27, p = 0.28). Surgeons reported generally more strain episodes during the first and second third of the operations; except in vascular operations, where no phase was associated with significantly higher strain levels, and emergency/trauma surgery, where strain episodes occurred primarily during the first third of the operation. Other professional groups showed different strain time patterns.

**Conclusions:**

Members of the OR teams experience strain differently across the phases of an operation. Thus, phases with high concentration requirements may highly vary across OR team members and no single phase of an operation can be defined as a “sterile cockpit” phase for all team members.

## Background

OR team members of all professions experience strain as a consequence of higher workload or stressors related to the task, team or environment. Strain can ensue from workload (defined as external demands), demands on the working memory or mental effort during task execution [1], or from social stressors and interpersonal tensions [2–4]. The term *strain* describes experienced stress [5] and the response to stressors [6]. For OR team members, strain can emerge from multiple sources, including task, collaboration, technology, ambiguity and patient care related time pressure [7, 8]. It is crucial to identify phases of high strain in the OR because strain through high workload or stress was found to decrease surgical technical performance, non technical skills and to impair anesthesiologists’ perceptions of relevant information [9–12]

Because strain is influenced by tasks and interactions in OR teams, strain is not constant during surgery. Phases of high strain may be followed by routine, low strain phases. Some of the more straining phases may be prospectively known, but others depend on the actual situation and events happening during the operation [13]. Identifying *when* phases of high workload occur during operations was a crucial step in the definition of phases of the operations during which interruptions and distractions should be minimized. Such phases are often described as sterile cockpit phases, or no interruption zones and were imported from aviation to the medical domain [14]. However, a major barrier to such intervention is that workload phases differ for different members of the OR team. In a smaller scale cardiac surgery study, anesthesia providers and circulating nurses reported high workload before, at the begin and towards the end of the surgery, whereas surgeons, and to a lesser extent scrub technicians, were most stressed during the middle, surgical repair phase of cardiac surgeries [15]. These studies suggest that phases of high strain may not be the same and do not have the same intensity for the different members of the surgical team. Because phases of high strain are closely coupled to task demands, they may also be different for different surgical procedures.

To our knowledge, besides of the study of Wadhera and colleagues [15], which was based on a small sample and a single surgical type, a systematic study to map intensity of strain across operative phases for all OR team members has not yet been undertaken. Whereas most research is based on the assumption that strain phases occur simultaneously for all team members, we know little about the temporal patterns of strain episodes for different OR team members. The aim of the present study was to identify phases of high strain for different professional groups working in the operating room (surgeons, surgery residents, anesthesiologists, scrub technicians, circulating nurses and medical students) and to compare those phases of high strain for different types of surgeries.

## Methods

### Study design and setting

The data were collected as part of a broader study on tense experiences in the OR. We conducted a prospective, observational, guided recall study, collecting OR team members’ experiences of strain immediately after surgical procedures at one 700-bed rural hospital in North America. Data were collected over a six-month period from 2018 to 2019. We also conducted explorative interviews with surgeons. The study was approved by the Institutional Review Board of Carilion Clinic (#2524). Prior to data collection, all participants were informed about the study and their right to refuse to participate via e-mail; verbal informed consent was obtained from all participants at data collection.

### Sample

The sample consisted of 113 operations performed in five surgical departments (pediatric, general, vascular and trauma/emergency general surgery as well as gynecology). Twenty-four different surgeons led these operations. Sixteen of these surgeons (three to four surgeons from each department) were interviewed.

The typical team composition during operations included a surgeon, a surgical resident, a scrub technician, a circulating nurse, an anesthesiologist or CRNA (certified registered nurse anesthetist) and for some operations a medical student. All teams were informed about the general project and type of data collection prior to study start. Study participants were blinded as to the aims of this study, and performed their work as normal.

All team members present were invited to participate in the guided recall immediately after the operations included in the study.

### Measures

#### Post-surgery guided recall

Based on previous work to assess affect over time in diverse settings [16], we developed a guided recall method to assess experienced strain during the operations. The guided recall was integrated into a short paper and pencil questionnaire.

**Fig. 1:**
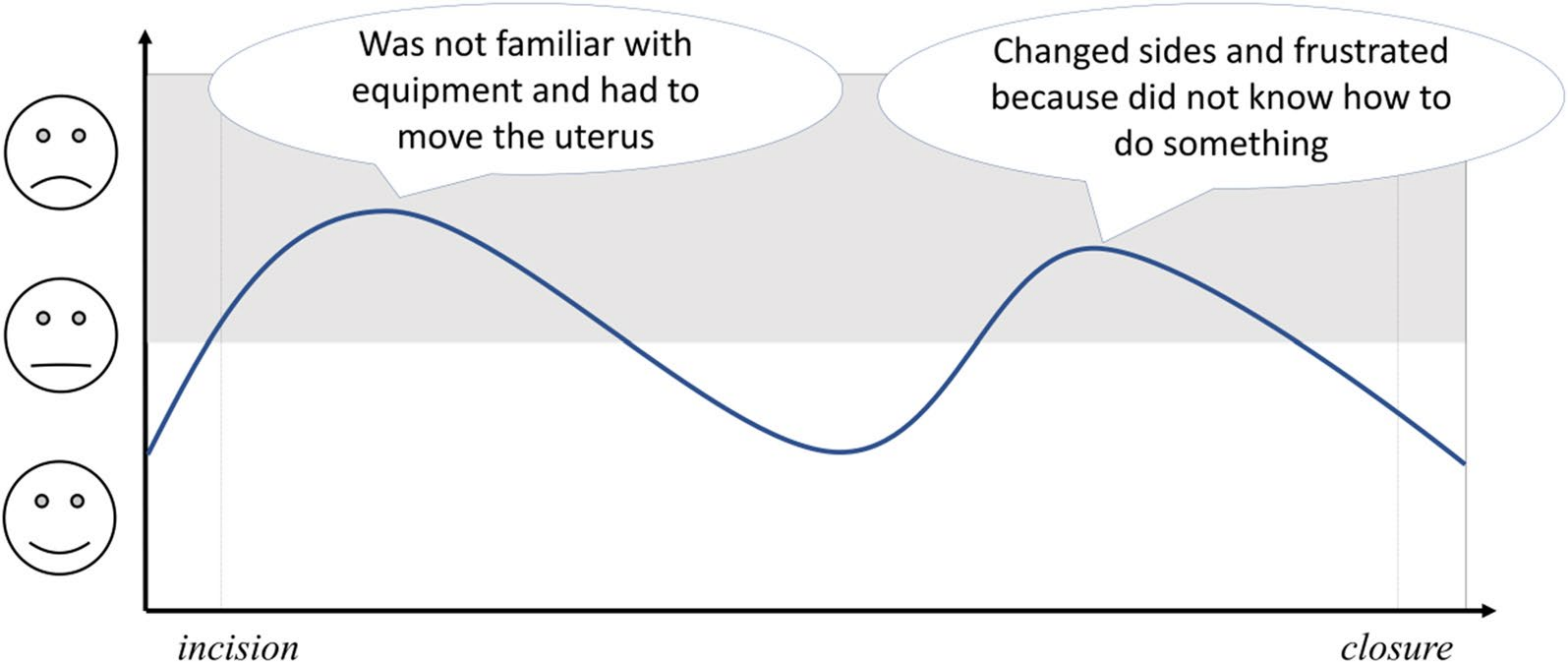
Guided recall tool to measure strain during operations and examples of strain episodes

Immediately after an operation, a researcher with a background in psychology (SK) invited each team member individually to draw a line representing the strain moments they experienced during the operation. The researcher explained that strain included all tense moments experienced, independent of their source. The time frame depicted the duration of the operation, ranging from shortly before incision to the end of the operation (see Fig. 1 for an example of two strain phases reported by junior surgical team members during gynecological operations). The drawing was two-dimensional, with the operation timeline on the x-axis and the strain level on the y-axis. Smiley faces were used on the y-axis to represent strain, as a simple alternative to Likert scale format [17]. Team members were also invited to describe the nature of each tense moment. Responses were collected confidentially, and team members were blind to the responses of the rest of the team.

#### Guided-recall data preparation

We translated the drawings of each participant into numerical values, using the peak of the curve and the slope of the line towards the peak. We then defined five phases of the operations based on the drawings: a phase before incision, the first third, the second third, the last third of the intra-operative time and the phase after closure. We applied the same definition for each drawing, and the three phases between incision and closure were each calculated as one third of the distance between the incision and closure mark on the drawing (in centimeters). For each top of the curve—highest point on a curve pointing to a strain moment, independently of its height on the y-axis, we identified in which phase of the operation it occurred, based on the x-axis timeline of the graph. For each team member, we calculated the total number of strain episodes per phase of the operation.

### Statistical analysis

To analyze the data, we used General Linear Modeling (GLM) with repeated measures. In the initial GLM model, we first compared strain experienced by all team members (overall) across the different phases of the operations. We used GLM to analyze variation of strain levels during the different temporal phases of the operation overall, within professional groups, between professional groups, and between the different types of operations. Our dependent variable was the number of strain episodes reported. Post-hoc tests based on least significant differences (LSD) were conducted to identify differences across different phases of the operation for each professional group. With univariate ANOVAs, we compared, for each phase of the surgery, the frequency of strain episodes across professional groups, independently of the surgery type. Significant differences across professional groups were calculated based on LSD post hoc tests.

p values less than 0.05 were considered statistically significant. The statistics were performed with IBM SPSS Statistics for Windows, version 25.

#### Interviews

The explorative interviews were conducted in a one-to-one setting by one researcher (SK) and were semi-structured [18]. The interview questions related to triggers of strain episodes in the OR, and specifically when higher strain episodes were experienced during an operation. They did not refer to single operations, but to the general experiences of a participant. The interviews were audio-recorded and transcribed. We performed a content analysis to identify phases of the operation that were described as associated with higher strain levels.

## Results

### Descriptive results

We collected a total of 693 guided recalls from OR team members after the 113 operations included in the study, with a mean number of guided recalls per operation of 6. The overall mean duration of the operations was 78 min (SD = 62 min; Table 1). The number of participants for each profession and surgery type varied with response rates ranging from 93.8 to 97.3% (Table 2). The most frequent reason for missing responses was that a team member could not be asked to fill out the guided recall (e.g. because he/she left the OR early). Three individuals opted to not complete the guided recall.

**Table 1:** Number of operations included for each surgery type and mean duration

Surgery type	n	Mean duration	SD duration
Pediatric	23	49.09	40.34
Gynecology	23	109.43	92.31
General surgery	22	82.64	59.98
Trauma/emergency	23	82.30	44.19
Vascular	22	68.14	44.03
Total	113	78.37	61.78

**Table 2:** Number of study participants for each profession per surgery type

	Surgeons	Residents	Medical students	Scrub technicians	Circulating nurses	Anesthesiology specialists	Total
Pediatric	24	22	14	25	26	29	140
Gynecology	23	29	15	25	33	20	145
General surgery	22	20	10	37	27	27	143
Trauma/emergency	23	37	14	23	31	29	157
Vascular	23	12	0	26	26	21	108
Total^a^	115	120	53	136	143	126	693

^a^Some participants filled out a guided recall for several operations. Also, when more than one team member of a same professional group was present during an operation, all were invited to fill out a guided recall. This explains that the total number of participants exceeds the number of operations included, except for the medical students

### Strain events across different phases of the surgery

The 693 guided recalls collected contained a total of 452 strain events. Examples of strain episodes reported included clinical, interpersonal, and systemic aspects (e.g. difficult dissection of a hernia sac, too busy to make sure that everything is correct, unsure what to do or noise in the hallway during induction). No strain episode was reported for the phase of the operation immediately after wound closure. For the rest of this analysis, we excluded this phase of the operation and included only four phases: the time before incision (phase 1), the first third of the operation (phase 2), the second third of the operation (phase 3) and the last third of the operation (phase 4). Frequency, means and standard deviation of strain events for each phase of the operation are displayed in Table 3. The frequency of strain varied significantly across the four phases of the operation (F = 52.75, p < 0.001; F = 7.29, p = 0.007, respectively quadratic and cubic effects). Strain was most frequently reported in the middle third of the operation (phase 3), followed by the first third of the operation (Phase 2) (see Additional file 1 for the detail of the post hoc tests).

**Table 3:** Overall strain episodes reported in the different phases of the operations

	n	Minimum	Maximum	Mean	SD
Before incision	89	0.00	5.00	0.79	1.03
First third	138	0.00	7.00	1.22	1.42
Second third	168	0.00	8.00	1.49	1.64
Last third	57	0.00	3.00	0.50	0.71
Total	452				

N = 113 operations

In a GLM model, we tested the effects of the phase of the operation, the professional group and the type of operation on experienced strain episodes. Results of within subjects models revealed a significant effect of the phase of the operation on the frequency of strain episodes [quadratic (F = 47.85, p < 0.001) and cubic (F = 8.94, p = 0.003) effects] (Additional file 2). These effects varied depending on the professional group [linear (F = 4.14, p = 0.001) and quadratic (F = 14.28, p < 0.001) effects]. This indicates that the different professional groups did not experience strain episodes during the same phases of the operation. The strain time patterns of the professional groups also varied depending on the surgery type (F = 4.92, p = 0.001, only cubic effects), suggesting that different surgical types show a different temporal pattern of strain. However, overall strain during different types of operations was similar (F = 1.27, p = 0.280, see Additional file 3 for between subject effects).

**Fig. 2:**
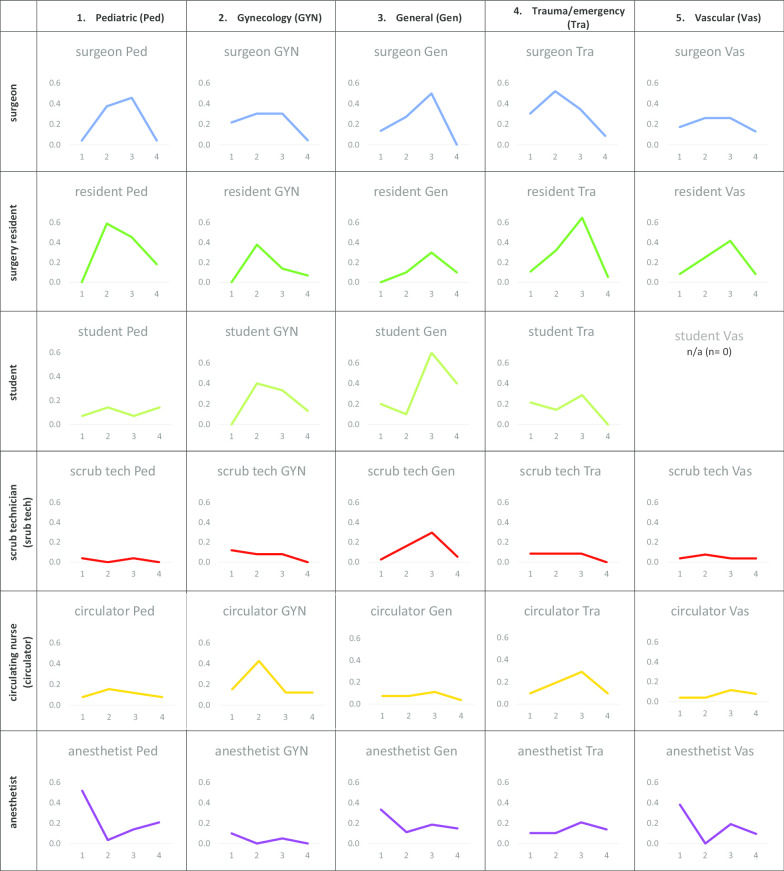
Mean frequency of strain events reported during the four phases of the operations. Note: x-axis: 1 = Phase 1, before incision, 2 = Phase 2, begin (first third of the operation), 3 = Phase 3, middle (middle third of the operation), 4 = Phase 4, end (last third of the operation); y-axis: scale representing mean frequency of strain episodes reported by the participants

Mean strain episodes per phase of the operations and significant differences are presented in Table 4 and illustrated in Fig. 2; the complete post hoc tests are presented in Additional file 4. We present some examples based on the statistically significant differences revealed by post-hoc tests based on LSD (least significant differences).

**Table 4:** Means and differences of experienced strain by the different professional groups, for each type of surgery

Professional groups	Surgical specialty^a^	N^b^	Phase 1		Phase 2		Phase 3		Phase 4		Differences across phases^c^
			M	SD	M	SD	M	SD	M	SD	
Surgeon	Ped	24	0.04	0.20	0.38	0.58	0.46	0.66	0.04	0.20	2 > 1, 2 > 4, 3 > 1, 3 > 4
	Gyn	23	0.22	0.42	0.30	0.47	0.30	0.47	0.04	0.21	2 > 4, 3 > 4
	Gen	22	0.14	0.35	0.27	0.46	0.50	0.67	0.00	0.00	2 > 4, 3 > 4
	Tra	23	0.30	0.47	0.52	0.59	0.35	0.49	0.09	0.29	2 > 4
	Vas	23	0.17	0.39	0.26	0.45	0.26	0.45	0.13	0.34	
	All types	115	0.17	0.38	0.35	0.51	0.37	0.55	0.06	0.24	
Resident	Ped	22	0.00	0.00	0.59	0.50	0.45	0.67	0.18	0.39	2 > 1, 2 > 4, 3 > 1, 4 > 1
	Gyn	29	0.00	0.00	0.38	0.73	0.14	0.35	0.07	0.26	2 > 1, 2 > 4, 3 > 1
	Gen	20	0.00	0.00	0.10	0.31	0.30	0.47	0.10	0.31	3 > 1
	Tra	37	0.11	0.31	0.32	0.58	0.65	0.54	0.05	0.23	2 > 4, 3 > 1, 3 > 2, 3 > 4
	Vas	12	0.08	0.29	0.25	0.62	0.42	0.51	0.08	0.29	3 > 4
	All types	120	0.04	0.20	0.34	0.59	0.41	0.54	0.09	0.29	
Student	Ped	14	0.07	0.27	0.14	0.36	0.07	0.27	0.14	0.36	
	Gyn	15	0.00	0.00	0.40	0.51	0.33	0.62	0.13	0.35	2 > 1
	Gen	10	0.20	0.42	0.10	0.32	0.70	0.67	0.40	0.52	3 > 2
	Tra	14	0.21	0.43	0.14	0.36	0.29	0.61	0.00	0.00	
	Vas	0									
	All types	53	0.11	0.32	0.21	0.41	0.32	0.58	0.15	0.36	
Scrub tech	Ped	25	0.04	0.20	0.00	0.00	0.04	0.20	0.00	0.00	
	Gyn	25	0.12	0.33	0.08	0.28	0.08	0.28	0.00	0.00	
	Gen	37	0.03	0.16	0.16	0.44	0.30	0.52	0.05	0.23	3 > 1, 3 > 4
	Tra	23	0.09	0.29	0.09	0.29	0.09	0.29	0.00	0.00	
	Vas	26	0.04	0.20	0.08	0.27	0.04	0.20	0.04	0.20	
	All types	136	0.09	0.31	0.09	0.31	0.13	0.35	0.02	0.15	
Circulator	Ped	26	0.08	0.27	0.15	0.37	0.12	0.43	0.08	0.27	
	Gyn	33	0.15	0.36	0.42	0.50	0.12	0.42	0.12	0.33	2 > 1, 2 > 3, 2 > 4
	Gen	27	0.07	0.27	0.07	0.27	0.11	0.32	0.04	0.19	
	Tra	31	0.10	0.30	0.19	0.40	0.29	0.46	0.10	0.30	3 > 4
	Vas	26	0.04	0.20	0.04	0.20	0.12	0.33	0.08	0.27	
	All types	143	0.19	0.39	0.19	0.39	0.15	0.40	0.08	0.28	
Anesthesiologist	Ped	29	0.52	0.51	0.03	0.19	0.14	0.35	0.21	0.41	1 > 2, 1 > 3, 1 > 4
	Gyn	20	0.10	0.31	0.00	0.00	0.05	0.22	0.00	0.00	
	Gen	27	0.33	0.55	0.11	0.32	0.19	0.40	0.15	0.36	
	Tra	29	0.10	0.31	0.10	0.31	0.21	0.41	0.14	0.35	
	Vas	21	0.38	0.50	0.00	0.00	0.19	0.51	0.10	0.30	1 > 2, 1 > 4
	All types	126	0.16	0.39	0.06	0.23	0.16	0.39	0.13	0.33	
All professional groups and surgery types		693	0.13	0.34	0.20	0.43	0.24	0.47	0.08	0.27	
Differences across professional groups (independently of surgery type)^d^			Surg > Res Surg > Scrub Surg > Circul Anesth > Surg Anesth > Res Anesth > Stud Anesth > Scrub Anesth < Circul		Surg > Stud Surg > Scrub Surg > Circul Surg > Anesth Res > Scrub Res > Circul Res > Anesth Stu > Anesth Circul > Scrub Circul > Anesth		Surg > Scrub Surg > Circul Surg > Anesth Res > Scrub Res > Circul Res > Anesth Stud > Scrub Stud > Circul Stud > Anesth		Res > Scrub Stud > Surg Stud > Scrub Anesth > Scrub		

^a^Ped = pediatric, Gyn = gynecology, Gen = general surgery, Tra = trauma/emergency, Vas = vascular

^b^N = number of participants

^c^Statistically significant differences across phases, identified based on LSD post hoc test of GLM model; 1 = phase 1 (before incision), 2 = phase 2 (first third of the operation), 3 = phase 3 (middle third of the operation, 4 = phase 4 (last third of the operation); > means more strain in one phase compared to another phase of the operation

^d^Results based on LSD post hoc tests of a univariate ANOVA; Surg = surgeon, Res = resident, Stud = student, Scrub = scrub technician, Circul = circulating nurse

Surgeons reported generally more strain episodes during the first and second third of the operation (phases 2 and 3). Exceptions were vascular surgery, where no phase was associated with significantly higher strain levels, and trauma/emergency surgery, where strain episodes where concentrated during the first third of the operation. Residents showed similar patterns. However, residents experienced mostly strain episodes during the middle part of the operation (phase 3), in particular in trauma/emergency surgery. Interestingly, scrub technicians experienced more frequent strain episodes in the middle part of general surgeries (phase 3), whereas circulating nurses experienced more strain in the first third of gynecology operations (phase 2) and middle third of trauma/emergency operations (phase 3). Anesthetists reported more strain episodes only in the first phase of paediatric and vascular operations, i.e. during induction. Of note, there was a non-significant trend towards more strain during the last phase of pediatric operations (p = 0.057) (see Additional file 4) but not for other surgery types.

Differences across professional groups separately for each phase, independent of surgical type (see bottom part of Table 4), showed that anesthesiologists experienced more strain episodes in the phase before incision (i.e. induction) than most other professional groups. Surgeons, residents and students were more likely to experience strain episodes in phase 2 and 3. Medical students and residents experienced higher strain than other professional groups towards the end of the operation (see Additional file 5 for the detail of the post hoc tests).

### Results from the qualitative interviews

**Fig. 3:**
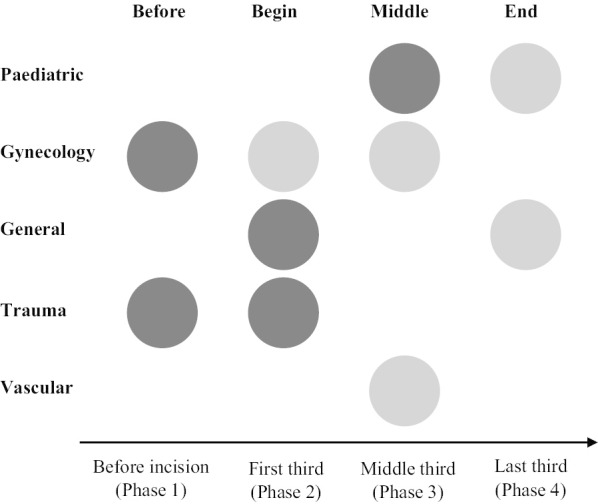
Graphical representation of the strain phases mentioned by surgeons from different surgical specialties in interviews

The qualitative interviews revealed that the surgeons could identify phases of the operation associated with higher strain levels. A synthesis of the results (Fig. 3) showed that surgeons from different surgical specialties identified different phases of their operations as related to an expected increase in strain.

The *phases immediately before the operation and the start of the operation* were mentioned as associated with high strain by the gynecology and the trauma/emergency surgeons. The reason for more strain was the particularly important preparation and organization of the material before starting the surgery. The *first phase* was described as particularly straining again by the trauma/emergency surgeons, gynecology and, to a somewhat lesser extent, by general surgeons. Trauma surgeons mentioned the uncertainty and decision making as an important challenge in the first phase, because detailed planning of the surgery was not possible beforehand. A general surgeon also mentioned that getting everyone’s attention for the safety checks at the start of the operation as a critical part of the process. The *middle phase* of the operation was mentioned to be associated with higher strain levels only by the pediatric, gynecology and vascular surgeons mainly due to higher technical demands. The *end of the operation* was not mentioned as a high strain phase with the exception of pediatrics and general surgeons; reasons for strain in this phase were an increased level of distractions and organizational challenges associated with patient transfer to the post operative care unit.

## Discussion

Our results showed that strain episodes experienced by OR team members varied as a function of the phase of the operation, professional group, and of the surgical specialty. Overall, the guided recall and the interview results converged well. Thus, professional groups in the OR experience high strain in different phases of the operation even within the same surgical type. In addition, phases of strain vary across surgical types. We can thus refute a general phase model of strain for all surgeries or across different professions.

### Different professional groups experience different strain phases

Consistent with previous research [19], in this study, *surgeons* showed overall more strain than all other team members during most phases of the operation. Across all surgeries, surgeons reported higher strain levels during the middle part of the surgeries, as shown in previous studies [15, 20]. However, when types of surgeries were considered, the pattern was more complex. Trauma surgeons reported higher strain levels at the beginning, mentioning organization of resources, uncertainty about the operation and decisions to be made, in line with previous research that showed that novel situations are likely to trigger uncertainty in surgeons [21]. For most of the surgical specialties, the guided recall and interview data coincide. However, the general surgeons’ guided recalls showed a clear peak of strain during the middle third of the surgeries, whereas in the interview, the surgeons labeled the first and last third of the surgery as straining. Interestingly, the reasons given in the interviews included organizational constraints, teamwork and distractions as predictors of strain.

*Residents’* reports of more frequent strain phases only partly mirrored the surgeons’ strain patterns. For most operations, residents reported more strain in the middle third of the operation. Again, this coincides with the phase of highest task demands, because in the middle third, residents often operate actively under the supervision of the surgeon. This was particularly the case in trauma/emergency surgery. Similarly, *medical students* reported more strain during the phases in which they were more actively involved in the operation and collaborating closer with the team (e.g. during the last phase of the operation, when they help closing the wound).

For most operations, our results showed less frequent strain for the *nurses (scrub technicians and circulators)*. Previous research had identified the phase before the start of the surgery (e.g. material preparation) [22] and the end of the operation [15] as particularly straining for nurses, because the phases of preparation and the final counts are critical tasks. Our results where more differentiated and again revealed different phase patterns for different procedures: During general surgery procedures, scrub technicians showed higher levels of strain in the middle phase, mirroring the pattern of the surgeons and potentially due to the particularly high number of instruments in general surgery.

*Circulating nurses* showed higher amount of strain in the first third of the operation in gynecology operations, probably because of the complex organization of the material, mentioned by the surgeons in the interviews and found to be a major source of interruptions, occurring mostly in the first thirty minutes of gynecology operations [23]. In trauma/emergency surgeries, they reported more strain in the middle third, potentially because they had to prepare new technical equipment after the operative decisions were made by the surgeons.

### Anesthetists

Most previous studies found the induction and emergence phases related to strain for the anesthetists and parallels were made with take-off and landing in aviation [15, 24, 25]. Our results were more differentiated. We found that only induction (the first phase) but not emergence, was associated with more strain. Pediatric surgery procedures were an exception; the last phase of the surgery, emergence from anesthesia, was associated with more strain, albeit only as a statistical trend. Also, strain levels at induction depended on the type of surgery, with higher strain at induction particularly for pediatric and vascular surgery patients. An explanation could be that those patients require particularly complex work processes.

Overall, this study showed that often made assumptions do not hold: The different members of the surgical team did not experience high strain at the same time during the same procedure. In addition, there is no overall temporal pattern of strain even within a profession, because the surgical type heavily influences strain patterns.

#### Guided reports to measure strain

The data were collected immediately after surgeries. This enhances the external validity of the study, as previous studies on this topic were often based on simulations, did not compare different professional groups [1], or were based on general questionnaires [15]. The methodology allowed to capture strain episodes emanating from different sources (cognitive, emotional, teamwork-related). Specific questionnaires often concentrate on one source of strain. For example, the NASA-TLX or SURG-TLX tool specifically measure cognitive workload [1]. Collecting the data immediately after the operation helped minimize recall and memory biases often associated with self-report [26]; it also allowed to capture strain related to stress and negative emotions experienced by OR teams and detrimental for surgical performance [11]. The results from the explorative interviews mostly coincided with the results of the guided recalls and offered additional information to interpret the data.

Our study also has limitations. The operations included were performed in a single center and operations performed within a same department were relatively heterogenous. A replication and finer distinction of surgical type in several centers would allow to show more diverse patterns, but also to disentangle strain related to the task and influencing factors associated with the organization, resources, and staffing. Further, we relied solely on reports of the participants. New technological developments allow to measure strain in terms of physiological changes over time, in particular in relation to the stress experienced by the OR teams (e.g. heart rate variability) [1, 9]. Although, these new technologies are not likely to replace data collected based on subjective experiences of OR team members, they may measure complementary facets of strain [27].

## Conclusions

Our results support the claim of previous researchers to better take into account task requirements of different professional groups [15, 28, 29] and the types of surgical procedure [12] when analyzing strain of surgical team members. A better awareness of these complex patterns may also inform interventions to reduce distractions and interruptions, particularly during critical moments [25], during which workload should not unnecessarily be added [30].

## Supplementary information

**Additional file 1.** Post hoc test showing differences across phases of the operations over all professions and all operation types.

**Additional file 2.** Results of the GLM Model for within subjects effects.

**Additional file 3.** Results of the GLM Model for between subjects model.

**Additional file 4.** Post hoc tests from the GLM model comparing differences across phases of the surgery for different professions and operation types.

**Additional file 5.** Post-hoc tests from Univariate Anovas comparing frequency of strain reported by the different professions, across all types of operations.

## Data Availability

The datasets generated and analyzed during the current study are not publicly available to protect the confidentiality of the participants but are available from the corresponding author on reasonable request and approval of the ethics committee of Carilion Clinic.

## Abbreviations

OR: Operating room
GLM: General linear modeling
ANOVA: Analysis of variance
