# Supplementary material for: Episodes of strain experienced in the operating room: impact of the type of surgery, the profession and the phase of the operation
Source: BMC Surg. 2020 Dec 7;20:318. doi: 10.1186/s12893-020-00937-y (PMC7720529; doi:10.1186/s12893-020-00937-y)
Supplement: Supplementary file 1 — Additional file 1. Post hoc test showing differences across phases of the operations over all professions and all operation types. [file 12893_2020_937_MOESM1_ESM.pdf]

Additional File 1: Post hoc test showing differences across phases of the operations over all professions and all operation types

		Mean Difference (I-J)	SE	p value	95% Confidence Interval Lower Bound	Upper Bound
Phase 1	Phase 2	-0.07	0.02	0.001	-0.11	-0.03
	Phase 3	-0.11	0.02	0.000	-0.15	-0.07
	Phase 4	0.04	0.02	0.004	0.01	0.07
Phase 2	Phase 1	0.07	0.02	0.001	0.03	0.11
	Phase 3	-0.04	0.02	0.036	-0.08	0.00
	Phase 4	0.11	0.02	0.000	0.08	0.15
Phase 3	Phase 1	0.11	0.02	0.000	0.07	0.15
	Phase 2	0.04	0.02	0.036	0.00	0.08
	Phase 4	0.16	0.02	0.000	0.12	0.19
Phase 4	Phase 1	-0.04	0.02	0.004	-0.07	-0.01
	Phase 2	-0.11	0.02	0.000	-0.15	-0.08
	Phase 3	-0.16	0.02	0.000	-0.19	-0.12
