# Supplementary material for: Episodes of strain experienced in the operating room: impact of the type of surgery, the profession and the phase of the operation
Source: BMC Surg. 2020 Dec 7;20:318. doi: 10.1186/s12893-020-00937-y (PMC7720529; doi:10.1186/s12893-020-00937-y)
Supplement: Supplementary file 2 — Additional file 2. Results of the GLM Model for within subjects effects. [file 12893_2020_937_MOESM2_ESM.pdf]

Additional file 2: Results of the GLM Model for within subjects effects

	Linear effects			Quadratic effects			Cubic effects		
	Df	F	Sig	df	F	Sig	df	F	Sig
Phase[1]	1	1.09	0.298	1	47.85	0.000	1	8.94	0.003
Interaction term phase x professional group[2]	5	4.14	0.001	5	14.28	0.000	5	1.60	0.157
Interaction term phase x surgery type[3]	4	1.85	0.118	4	1.31	0.266	4	4.92	0.001
Interaction term phase x professional group x surgery type	19	1.58	0.055	19	1.59	0.054	19	1.66	0.039

Note.[1] Phase of the operation: immediately before incision, first third, middle third and last third of the operation; [2] Professional group: attending surgeon, resident in surgery, medical student, scrub technician, circulating nurse, anesthetist; [3] Surgery type: pediatric, gynecology, general, trauma/emergency and vascular surgery.
