# Supplementary material for: Episodes of strain experienced in the operating room: impact of the type of surgery, the profession and the phase of the operation
Source: BMC Surg. 2020 Dec 7;20:318. doi: 10.1186/s12893-020-00937-y (PMC7720529; doi:10.1186/s12893-020-00937-y)
Supplement: Supplementary file 3 — Additional file 3. Results of the GLM Model for between subjects model. [file 12893_2020_937_MOESM3_ESM.pdf]

Additional file 3: Results of the GLM Model for between subjects model

	df	F	p value
Intercept	1	438.25	0.000
Professional group	5	12.90	0.000
Surgery type	4	1.27	0.280
Interaction term professional group x surgery type	19	2.93	0.000
