# Supplementary material for: Episodes of strain experienced in the operating room: impact of the type of surgery, the profession and the phase of the operation
Source: BMC Surg. 2020 Dec 7;20:318. doi: 10.1186/s12893-020-00937-y (PMC7720529; doi:10.1186/s12893-020-00937-y)
Supplement: Supplementary file 4 — Additional file 4. Post hoc tests from the GLM model comparing differences across phases of the surgery for different professions and operation types. [file 12893_2020_937_MOESM4_ESM.pdf]

Additional file 4: Post hoc tests from the GLM model comparing differences across phases of the surgery for different professions and operation types

Profession	Surgery type	Phases of the operation[1]	Phases of the operation[1]		SE	p value	95% Confidence Interval Lower Bound	95% Confidence Interval Upper Bound
attending	pediatric	Ph 1	Ph 2	-0.33	0.13	0.017	-0.60	-0.06
			Ph 3	-0.42	0.15	0.009	-0.72	-0.11
			Ph 4	0.00	0.06	1.000	-0.12	0.12
		Ph 2	Ph 1	0.33	0.13	0.017	0.06	0.60
			Ph 3	-0.08	0.15	0.575	-0.39	0.22
			Ph 4	0.33	0.12	0.008	0.09	0.57
		Ph 3	Ph 1	0.42	0.15	0.009	0.11	0.72
			Ph 2	0.08	0.15	0.575	-0.22	0.39
			Ph 4	0.42	0.13	0.005	0.14	0.69
		Ph 4	Ph 1	0.00	0.06	1.000	-0.12	0.12
			Ph 2	-0.33	0.12	0.008	-0.57	-0.09
			Ph 3	-0.42	0.13	0.005	-0.69	-0.14
attending	gynecology	Ph 1	Ph 2	-0.09	0.11	0.426	-0.31	0.14
			Ph 3	-0.09	0.14	0.539	-0.38	0.20
			Ph 4	0.17	0.10	0.103	-0.04	0.39
		Ph 2	Ph 1	0.09	0.11	0.426	-0.14	0.31
			Ph 3	0.00	0.11	1.000	-0.23	0.23
			Ph 4	0.26	0.09	0.011	0.07	0.46
		Ph 3	Ph 1	0.09	0.14	0.539	-0.20	0.38
			Ph 2	0.00	0.11	1.000	-0.23	0.23
			Ph 4	0.26	0.11	0.030	0.03	0.49
		Ph 4	Ph 1	-0.17	0.10	0.103	-0.39	0.04
			Ph 2	-0.26	0.09	0.011	-0.46	-0.07
			Ph 3	-0.26	0.11	0.030	-0.49	-0.03
attending	general	Ph 1	Ph 2	-0.14	0.12	0.266	-0.38	0.11
			Ph 3	-0.36	0.17	0.042	-0.71	-0.01
			Ph 4	0.14	0.07	0.083	-0.02	0.29
		Ph 2	Ph 1	0.14	0.12	0.266	-0.11	0.38
			Ph 3	-0.23	0.20	0.261	-0.64	0.18
			Ph 4	0.27	0.10	0.011	0.07	0.47
		Ph 3	Ph 1	0.36	0.17	0.042	0.01	0.71
			Ph 2	0.23	0.20	0.261	-0.18	0.64
			Ph 4	0.50	0.14	0.002	0.20	0.80
		Ph 4	Ph 1	-0.14	0.07	0.083	-0.29	0.02
			Ph 2	-0.27	0.10	0.011	-0.47	-0.07
			Ph 3	-0.50	0.14	0.002	-0.80	-0.20
attending	trauma/emergency	Ph 1	Ph 2	-0.22	0.19	0.260	-0.61	0.17
			Ph 3	-0.04	0.17	0.803	-0.40	0.31
			Ph 4	0.22	0.11	0.057	-0.01	0.44

Additional file 4

		Ph 2	Ph 1	0.22	0.19	0.260	-0.17	0.61
			Ph 3	0.17	0.10	0.103	-0.04	0.39
			Ph 4	0.44	0.15	0.009	0.12	0.75
		Ph 3	Ph 1	0.04	0.17	0.803	-0.31	0.40
			Ph 2	-0.17	0.10	0.103	-0.39	0.04
			Ph 4	0.26	0.13	0.056	-0.01	0.53
		Ph 4	Ph 1	-0.22	0.11	0.057	-0.44	0.01
			Ph 2	-0.44	0.15	0.009	-0.75	-0.12
			Ph 3	-0.26	0.13	0.056	-0.53	0.01
attending	vascular	Ph 1	Ph 2	-0.09	0.11	0.426	-0.31	0.14
			Ph 3	-0.09	0.14	0.539	-0.38	0.20
			Ph 4	0.04	0.12	0.714	-0.20	0.29
		Ph 2	Ph 1	0.09	0.11	0.426	-0.14	0.31
			Ph 3	0.00	0.14	1.000	-0.29	0.29
			Ph 4	0.13	0.10	0.186	-0.07	0.33
		Ph 3	Ph 1	0.09	0.14	0.539	-0.20	0.38
			Ph 2	0.00	0.14	1.000	-0.29	0.29
			Ph 4	0.13	0.11	0.266	-0.11	0.37
		Ph 4	Ph 1	-0.04	0.12	0.714	-0.29	0.20
			Ph 2	-0.13	0.10	0.186	-0.33	0.07
			Ph 3	-0.13	0.11	0.266	-0.37	0.11
resident	pediatric	Ph 1	Ph 2	-0.59	0.11	0.000	-0.81	-0.37
			Ph 3	-0.46	0.14	0.005	-0.75	-0.16
			Ph 4	-0.18	0.08	0.042	-0.36	-0.01
		Ph 2	Ph 1	0.59	0.11	0.000	0.37	0.81
			Ph 3	0.14	0.18	0.451	-0.23	0.51
			Ph 4	0.41	0.14	0.009	0.11	0.70
		Ph 3	Ph 1	0.46	0.14	0.005	0.16	0.75
			Ph 2	-0.14	0.18	0.451	-0.51	0.23
			Ph 4	0.27	0.15	0.083	-0.04	0.58
		Ph 4	Ph 1	0.18	0.08	0.042	0.01	0.36
			Ph 2	-0.41	0.14	0.009	-0.70	-0.11
			Ph 3	-0.27	0.15	0.083	-0.58	0.04
resident	gynecology	Ph 1	Ph 2	-0.38	0.14	0.009	-0.66	-0.10
			Ph 3	-0.14	0.07	0.043	-0.27	0.00
			Ph 4	-0.07	0.05	0.161	-0.17	0.03
		Ph 2	Ph 1	0.38	0.14	0.009	0.10	0.66
			Ph 3	0.24	0.15	0.129	-0.07	0.56
			Ph 4	0.31	0.15	0.048	0.00	0.62
		Ph 3	Ph 1	0.14	0.07	0.043	0.00	0.27
			Ph 2	-0.24	0.15	0.129	-0.56	0.07
			Ph 4	0.07	0.08	0.424	-0.11	0.24
		Ph 4	Ph 1	0.07	0.05	0.161	-0.03	0.17
			Ph 2	-0.31	0.15	0.048	-0.62	0.00
			Ph 3	-0.07	0.08	0.424	-0.24	0.11
resident	general	Ph 1	Ph 2	-0.10	0.07	0.163	-0.24	0.04
			Ph 3	-0.30	0.11	0.010	-0.52	-0.08
			Ph 4	-0.10	0.07	0.163	-0.24	0.04
		Ph 2	Ph 1	0.10	0.07	0.163	-0.04	0.24

			Ph 3	-0.20	0.12	0.104	-0.44	0.04
			Ph 4	0.00	0.10	1.000	-0.21	0.21
		Ph 3	Ph 1	0.30	0.11	0.010	0.08	0.52
			Ph 2	0.20	0.12	0.104	-0.04	0.44
			Ph 4	0.20	0.14	0.163	-0.09	0.49
		Ph 4	Ph 1	0.10	0.07	0.163	-0.04	0.24
			Ph 2	0.00	0.10	1.000	-0.21	0.21
			Ph 3	-0.20	0.14	0.163	-0.49	0.09
resident	trauma/emergency	Ph 1	Ph 2	-0.22	0.12	0.073	-0.45	0.02
			Ph 3	-0.54	0.11	0.000	-0.76	-0.32
			Ph 4	0.05	0.07	0.422	-0.08	0.19
		Ph 2	Ph 1	0.22	0.12	0.073	-0.02	0.45
			Ph 3	-0.32	0.12	0.012	-0.57	-0.08
			Ph 4	0.27	0.10	0.010	0.07	0.47
		Ph 3	Ph 1	0.54	0.11	0.000	0.32	0.76
			Ph 2	0.32	0.12	0.012	0.08	0.57
			Ph 4	0.60	0.10	0.000	0.39	0.79
		Ph 4	Ph 1	-0.05	0.07	0.422	-0.19	0.08
			Ph 2	-0.27	0.10	0.010	-0.47	-0.07
			Ph 3	-0.60	0.10	0.000	-0.79	-0.39
resident	vascular	Ph 1	Ph 2	-0.17	0.21	0.438	-0.62	0.29
			Ph 3	-0.33	0.19	0.104	-0.75	0.08
			Ph 4	0.00	0.12	1.000	-0.27	0.27
		Ph 2	Ph 1	0.17	0.21	0.438	-0.29	0.62
			Ph 3	-0.17	0.21	0.438	-0.62	0.29
			Ph 4	0.17	0.21	0.438	-0.29	0.62
		Ph 3	Ph 1	0.33	0.19	0.104	-0.08	0.75
			Ph 2	0.17	0.21	0.438	-0.29	0.62
			Ph 4	0.33	0.14	0.039	0.02	0.65
		Ph 4	Ph 1	0.00	0.12	1.000	-0.27	0.27
			Ph 2	-0.17	0.21	0.438	-0.62	0.29
			Ph 3	-0.33	0.14	0.039	-0.65	-0.02
student	pediatric	Ph 1	Ph 2	-0.07	0.13	0.583	-0.35	0.20
			Ph 3	0.00	0.10	1.000	-0.23	0.23
			Ph 4	-0.07	0.13	0.583	-0.35	0.20
		Ph 2	Ph 1	0.07	0.13	0.583	-0.20	0.35
			Ph 3	0.07	0.13	0.583	-0.20	0.35
			Ph 4	0.00	0.10	1.000	-0.23	0.23
		Ph 3	Ph 1	0.00	0.10	1.000	-0.23	0.23
			Ph 2	-0.07	0.13	0.583	-0.35	0.20
			Ph 4	-0.07	0.07	0.336	-0.23	0.08
		Ph 4	Ph 1	0.07	0.13	0.583	-0.20	0.35
			Ph 2	0.00	0.10	1.000	-0.23	0.23
			Ph 3	0.07	0.07	0.336	-0.08	0.23
student	gynecology	Ph 1	Ph 2	-0.40	0.13	0.009	-0.68	-0.12
			Ph 3	-0.33	0.16	0.055	-0.68	0.01
			Ph 4	-0.13	0.09	0.164	-0.33	0.06
		Ph 2	Ph 1	0.40	0.13	0.009	0.12	0.68

Additional file 4

			Ph 3	0.07	0.15	0.670	-0.26	0.40
			Ph 4	0.27	0.15	0.104	-0.06	0.60
		Ph 3	Ph 1	0.33	0.16	0.055	-0.01	0.68
			Ph 2	-0.07	0.15	0.670	-0.40	0.26
			Ph 4	0.20	0.17	0.271	-0.17	0.57
		Ph 4	Ph 1	0.13	0.09	0.164	-0.06	0.33
			Ph 2	-0.27	0.15	0.104	-0.60	0.06
			Ph 3	-0.20	0.17	0.271	-0.57	0.17
student	general	Ph 1	Ph 2	0.10	0.18	0.591	-0.31	0.51
			Ph 3	-0.50	0.27	0.096	-1.11	0.11
			Ph 4	-0.20	0.13	0.168	-0.50	0.10
		Ph 2	Ph 1	-0.10	0.18	0.591	-0.51	0.31
			Ph 3	-0.60	0.16	0.005	-0.97	-0.23
			Ph 4	-0.30	0.21	0.193	-0.78	0.18
		Ph 3	Ph 1	0.50	0.27	0.096	-0.11	1.11
			Ph 2	0.60	0.16	0.005	0.23	0.97
			Ph 4	0.30	0.30	0.343	-0.38	0.98
		Ph 4	Ph 1	0.20	0.13	0.168	-0.10	0.50
			Ph 2	0.30	0.21	0.193	-0.18	0.78
			Ph 3	-0.30	0.30	0.343	-0.98	0.38
student	trauma/emergency	Ph 1	Ph 2	0.07	0.16	0.671	-0.28	0.43
			Ph 3	-0.07	0.22	0.752	-0.55	0.41
			Ph 4	0.21	0.11	0.082	-0.03	0.46
		Ph 2	Ph 1	-0.07	0.16	0.671	-0.43	0.28
			Ph 3	-0.14	0.21	0.500	-0.59	0.30
			Ph 4	0.14	0.10	0.165	-0.07	0.35
		Ph 3	Ph 1	0.07	0.22	0.752	-0.41	0.55
			Ph 2	0.14	0.21	0.500	-0.30	0.59
			Ph 4	0.29	0.16	0.104	-0.07	0.64
		Ph 4	Ph 1	-0.21	0.11	0.082	-0.46	0.03
			Ph 2	-0.14	0.10	0.165	-0.35	0.07
			Ph 3	-0.29	0.16	0.104	-0.64	0.07
scrub tech	Pediatric	Ph 1	Ph 2	0.04	0.04	0.327	-0.04	0.12
			Ph 3	0.00	0.06	1.000	-0.12	0.12
			Ph 4	0.04	0.04	0.327	-0.04	0.12
		Ph 2	Ph 1	-0.04	0.04	0.327	-0.12	0.04
			Ph 3	-0.04	0.04	0.327	-0.12	0.04
			Ph 4	0.00	0.00		0.00	0.00
		Ph 3	Ph 1	0.00	0.06	1.000	-0.12	0.12
			Ph 2	0.04	0.04	0.327	-0.04	0.12
			Ph 4	0.04	0.04	0.327	-0.04	0.12
		Ph 4	Ph 1	-0.04	0.04	0.327	-0.12	0.04
			Ph 2	0.00	0.00		0.00	0.00
			Ph 3	-0.04	0.04	0.327	-0.12	0.04
scrub tech	gynecology	Ph 1	Ph 2	0.04	0.09	0.664	-0.15	0.23
			Ph 3	0.04	0.09	0.664	-0.15	0.23
			Ph 4	0.12	0.07	0.083	-0.02	0.26
		Ph 2	Ph 1	-0.04	0.09	0.664	-0.23	0.15

Additional file 4

			Ph 3	0.00	0.06	1.000	-0.12	0.12
			Ph 4	0.08	0.06	0.161	-0.03	0.19
		Ph 3	Ph 1	-0.04	0.09	0.664	-0.23	0.15
			Ph 2	0.00	0.06	1.000	-0.12	0.12
			Ph 4	0.08	0.06	0.161	-0.03	0.19
		Ph 4	Ph 1	-0.12	0.07	0.083	-0.26	0.02
			Ph 2	-0.08	0.06	0.161	-0.19	0.03
			Ph 3	-0.08	0.06	0.161	-0.19	0.03
scrub tech	general	Ph 1	Ph 2	-0.14	0.08	0.096	-0.30	0.03
			Ph 3	-0.27	0.09	0.006	-0.46	-0.08
			Ph 4	-0.03	0.05	0.571	-0.12	0.07
		Ph 2	Ph 1	0.14	0.08	0.096	-0.03	0.30
			Ph 3	-0.14	0.12	0.257	-0.37	0.10
			Ph 4	0.11	0.08	0.160	-0.04	0.26
		Ph 3	Ph 1	0.27	0.09	0.006	0.08	0.46
			Ph 2	0.14	0.12	0.257	-0.10	0.37
			Ph 4	0.24	0.09	0.010	0.06	0.43
		Ph 4	Ph 1	0.03	0.05	0.571	-0.07	0.12
			Ph 2	-0.11	0.08	0.160	-0.26	0.04
			Ph 3	-0.24	0.09	0.010	-0.43	-0.06
scrub tech	trauma/emergency	Ph 1	Ph 2	0.00	0.09	1.000	-0.18	0.18
			Ph 3	0.00	0.09	1.000	-0.18	0.18
			Ph 4	0.09	0.06	0.162	-0.04	0.21
		Ph 2	Ph 1	0.00	0.09	1.000	-0.18	0.18
			Ph 3	0.00	0.06	1.000	-0.13	0.13
			Ph 4	0.09	0.06	0.162	-0.04	0.21
		Ph 3	Ph 1	0.00	0.09	1.000	-0.18	0.18
			Ph 2	0.00	0.06	1.000	-0.13	0.13
			Ph 4	0.09	0.06	0.162	-0.04	0.21
		Ph 4	Ph 1	-0.09	0.06	0.162	-0.21	0.04
			Ph 2	-0.09	0.06	0.162	-0.21	0.04
			Ph 3	-0.09	0.06	0.162	-0.21	0.04
scrub tech	vascular	Ph 1	Ph 2	-0.04	0.07	0.574	-0.18	0.10
			Ph 3	0.00	0.06	1.000	-0.11	0.11
			Ph 4	0.00	0.06	1.000	-0.11	0.11
		Ph 2	Ph 1	0.04	0.07	0.574	-0.10	0.18
			Ph 3	0.04	0.04	0.327	-0.04	0.12
			Ph 4	0.04	0.07	0.574	-0.10	0.18
		Ph 3	Ph 1	0.00	0.06	1.000	-0.11	0.11
			Ph 2	-0.04	0.04	0.327	-0.12	0.04
			Ph 4	0.00	0.06	1.000	-0.11	0.11
		Ph 4	Ph 1	0.00	0.06	1.000	-0.11	0.11
			Ph 2	-0.04	0.07	0.574	-0.18	0.10
			Ph 3	0.00	0.06	1.000	-0.11	0.11
circulator	pediatric	Ph 1	Ph 2	-0.08	0.09	0.425	-0.27	0.12
			Ph 3	-0.04	0.10	0.713	-0.25	0.17
			Ph 4	0.00	0.06	1.000	-0.11	0.11
		Ph 2	Ph 1	0.08	0.09	0.425	-0.12	0.27

			Ph 3	0.04	0.09	0.664	-0.14	0.22
			Ph 4	0.08	0.09	0.425	-0.12	0.27
		Ph 3	Ph 1	0.04	0.10	0.713	-0.17	0.25
			Ph 2	-0.04	0.09	0.664	-0.22	0.14
			Ph 4	0.04	0.10	0.713	-0.17	0.25
		Ph 4	Ph 1	0.00	0.06	1.000	-0.11	0.11
			Ph 2	-0.08	0.09	0.425	-0.27	0.12
			Ph 3	-0.04	0.10	0.713	-0.25	0.17
circulator	gynecology	Ph 1	Ph 2	-0.27	0.12	0.027	-0.51	-0.03
			Ph 3	0.03	0.10	0.768	-0.18	0.24
			Ph 4	0.03	0.09	0.744	-0.16	0.22
		Ph 2	Ph 1	0.27	0.12	0.027	0.03	0.51
			Ph 3	0.30	0.10	0.006	0.10	0.51
			Ph 4	0.30	0.08	0.001	0.14	0.47
		Ph 3	Ph 1	-0.03	0.10	0.768	-0.24	0.18
			Ph 2	-0.30	0.10	0.006	-0.51	-0.10
			Ph 4	0.00	0.10	1.000	-0.20	0.20
		Ph 4	Ph 1	-0.03	0.09	0.744	-0.22	0.16
			Ph 2	-0.30	0.08	0.001	-0.47	-0.14
			Ph 3	0.00	0.10	1.000	-0.20	0.20
circulator	general	Ph 1	Ph 2	0.00	0.08	1.000	-0.16	0.16
			Ph 3	-0.04	0.06	0.574	-0.17	0.10
			Ph 4	0.04	0.06	0.574	-0.10	0.17
		Ph 2	Ph 1	0.00	0.08	1.000	-0.16	0.16
			Ph 3	-0.04	0.08	0.663	-0.21	0.14
			Ph 4	0.04	0.06	0.574	-0.10	0.17
		Ph 3	Ph 1	0.04	0.06	0.574	-0.10	0.17
			Ph 2	0.04	0.08	0.663	-0.14	0.21
			Ph 4	0.07	0.07	0.327	-0.08	0.23
		Ph 4	Ph 1	-0.04	0.06	0.574	-0.17	0.10
			Ph 2	-0.04	0.06	0.574	-0.17	0.10
			Ph 3	-0.07	0.07	0.327	-0.23	0.08
circulator	trauma/emergency	Ph 1	Ph 2	-0.10	0.10	0.325	-0.29	0.10
			Ph 3	-0.19	0.11	0.083	-0.41	0.03
			Ph 4	0.00	0.08	1.000	-0.16	0.16
		Ph 2	Ph 1	0.10	0.10	0.325	-0.10	0.29
			Ph 3	-0.10	0.10	0.325	-0.29	0.10
			Ph 4	0.10	0.08	0.264	-0.08	0.27
		Ph 3	Ph 1	0.19	0.11	0.083	-0.03	0.41
			Ph 2	0.10	0.10	0.325	-0.10	0.29
			Ph 4	0.19	0.09	0.031	0.02	0.37
		Ph 4	Ph 1	0.00	0.08	1.000	-0.16	0.16
			Ph 2	-0.10	0.08	0.264	-0.27	0.08
			Ph 3	-0.19	0.09	0.031	-0.37	-0.02
circulator	vascular	Ph 1	Ph 2	0.00	0.06	1.000	-0.11	0.11
			Ph 3	-0.08	0.08	0.327	-0.24	0.08
			Ph 4	-0.04	0.07	0.574	-0.18	0.10
		Ph 2	Ph 1	0.00	0.06	1.000	-0.11	0.11

			Ph 3	-0.08	0.05	0.161	-0.19	0.03
			Ph 4	-0.04	0.07	0.574	-0.18	0.10
		Ph 3	Ph 1	0.08	0.08	0.327	-0.08	0.24
			Ph 2	0.08	0.05	0.161	-0.03	0.19
			Ph 4	0.04	0.07	0.574	-0.10	0.18
		Ph 4	Ph 1	0.04	0.07	0.574	-0.10	0.18
			Ph 2	0.04	0.07	0.574	-0.10	0.18
			Ph 3	-0.04	0.07	0.574	-0.18	0.10
anesthetist	pediatric	Ph 1	Ph 2	0.48	0.09	0.000	0.29	0.68
			Ph 3	0.38	0.10	0.001	0.17	0.59
			Ph 4	0.31	0.11	0.010	0.08	0.54
		Ph 2	Ph 1	-0.48	0.09	0.000	-0.68	-0.29
			Ph 3	-0.10	0.06	0.083	-0.22	0.01
			Ph 4	-0.17	0.09	0.057	-0.35	0.01
		Ph 3	Ph 1	-0.38	0.10	0.001	-0.59	-0.17
			Ph 2	0.10	0.06	0.083	-0.01	0.22
			Ph 4	-0.07	0.11	0.537	-0.29	0.16
		Ph 4	Ph 1	-0.31	0.11	0.010	-0.54	-0.08
			Ph 2	0.17	0.09	0.057	-0.01	0.35
			Ph 3	0.07	0.11	0.537	-0.16	0.29
anesthetist	gynecology	Ph 1	Ph 2	0.10	0.07	0.163	-0.04	0.24
			Ph 3	0.05	0.05	0.330	-0.05	0.15
			Ph 4	0.10	0.07	0.163	-0.04	0.24
		Ph 2	Ph 1	-0.10	0.07	0.163	-0.24	0.04
			Ph 3	-0.05	0.05	0.330	-0.15	0.05
			Ph 4	0.00	0.00		0.00	0.00
		Ph 3	Ph 1	-0.05	0.05	0.330	-0.15	0.05
			Ph 2	0.05	0.05	0.330	-0.05	0.15
			Ph 4	0.05	0.05	0.330	-0.05	0.15
		Ph 4	Ph 1	-0.10	0.07	0.163	-0.24	0.04
			Ph 2	0.00	0.00		0.00	0.00
			Ph 3	-0.05	0.05	0.330	-0.15	0.05
anesthetist	general	Ph 1	Ph 2	0.22	0.13	0.110	-0.05	0.50
			Ph 3	0.15	0.14	0.294	-0.14	0.43
			Ph 4	0.19	0.11	0.096	-0.04	0.41
		Ph 2	Ph 1	-0.22	0.13	0.110	-0.50	0.05
			Ph 3	-0.07	0.07	0.327	-0.23	0.08
			Ph 4	-0.04	0.08	0.663	-0.21	0.14
		Ph 3	Ph 1	-0.15	0.14	0.294	-0.43	0.14
			Ph 2	0.07	0.07	0.327	-0.08	0.23
			Ph 4	0.04	0.08	0.663	-0.14	0.21
		Ph 4	Ph 1	-0.19	0.11	0.096	-0.41	0.04
			Ph 2	0.04	0.08	0.663	-0.14	0.21
			Ph 3	-0.04	0.08	0.663	-0.21	0.14
anesthetist	trauma/emergency	Ph 1	Ph 2	0.00	0.09	1.000	-0.18	0.18
			Ph 3	-0.10	0.10	0.326	-0.32	0.11
			Ph 4	-0.03	0.09	0.712	-0.22	0.16
		Ph 2	Ph 1	0.00	0.09	1.000	-0.18	0.18

			Ph 3	-0.10	0.09	0.264	-0.29	0.08
			Ph 4	-0.03	0.08	0.663	-0.19	0.13
		Ph 3	Ph 1	0.10	0.10	0.326	-0.11	0.32
			Ph 2	0.10	0.09	0.264	-0.08	0.29
			Ph 4	0.07	0.11	0.537	-0.16	0.29
		Ph 4	Ph 1	0.03	0.09	0.712	-0.16	0.22
			Ph 2	0.03	0.08	0.663	-0.13	0.19
			Ph 3	-0.07	0.11	0.537	-0.29	0.16
anesthetist	vascular	Ph 1	Ph 2	0.38	0.11	0.002	0.15	0.61
			Ph 3	0.19	0.11	0.104	-0.04	0.42
			Ph 4	0.29	0.10	0.010	0.08	0.50
		Ph 2	Ph 1	-0.38	0.11	0.002	-0.61	-0.15
			Ph 3	-0.19	0.11	0.104	-0.42	0.04
			Ph 4	-0.10	0.07	0.162	-0.23	0.04
		Ph 3	Ph 1	-0.19	0.11	0.104	-0.42	0.04
			Ph 2	0.19	0.11	0.104	-0.04	0.42
			Ph 4	0.10	0.14	0.493	-0.19	0.38
		Ph 4	Ph 1	-0.29	0.10	0.010	-0.50	-0.08
			Ph 2	0.10	0.07	0.162	-0.04	0.23
			Ph 3	-0.10	0.14	0.493	-0.38	0.19

Note. [1] Ph 1 = phase 1 (before incision), Ph 2 = phase 2 (first third of the operation), Ph 3 = phase 3 (middle third of the operation, Ph 4 = phase 4 (last third of the operation)
