# Supplementary material for: Episodes of strain experienced in the operating room: impact of the type of surgery, the profession and the phase of the operation
Source: BMC Surg. 2020 Dec 7;20:318. doi: 10.1186/s12893-020-00937-y (PMC7720529; doi:10.1186/s12893-020-00937-y)
Supplement: Supplementary file 5 — Additional file 5. Post-hoc tests from Univariate Anovas comparing frequency of strain reported by the different professions, across all types of operations. [file 12893_2020_937_MOESM5_ESM.pdf]

Additional file 5: Post-hoc tests from Univariate Anovas comparing frequency of strain reported by the different professions, across all types of operations.

Phase 1: phase before incision

		Mean Difference (I-J)	Std. Error	Sig.	95% Confidence Interval	
					Lower Bound	Upper Bound
attending	resident	0.13	0.04	0.002	0.05	0.22
	student	0.06	0.05	0.266	-0.05	0.17
	scrub tech	0.12	0.04	0.006	0.03	0.20
	circulator	0.08	0.04	0.044	0.00	0.16
	anesthetist	-0.12	0.04	0.005	-0.20	-0.04
resident	attending	-0.13	0.04	0.002	-0.22	-0.05
	student	-0.07	0.05	0.187	-0.18	0.03
	scrub tech	-0.02	0.04	0.677	-0.10	0.06
	circulator	-0.05	0.04	0.226	-0.13	0.03
	anesthetist	-0.25	0.04	0.000	-0.33	-0.17
student	attending	-0.06	0.05	0.266	-0.17	0.05
	resident	0.07	0.05	0.187	-0.03	0.18
	scrub tech	0.05	0.05	0.307	-0.05	0.16
	circulator	0.02	0.05	0.673	-0.08	0.13
	anesthetist	-0.18	0.05	0.001	-0.29	-0.07
scrub tech	attending	-0.12	0.04	0.006	-0.20	-0.03
	resident	0.02	0.04	0.677	-0.06	0.10
	student	-0.05	0.05	0.307	-0.16	0.05
	circulator	-0.03	0.04	0.415	-0.11	0.05
	anesthetist	-0.23	0.04	0.000	-0.31	-0.16
circulator	attending	-0.08	0.04	0.044	-0.16	0.00
	resident	0.05	0.04	0.226	-0.03	0.13
	student	-0.02	0.05	0.673	-0.13	0.08
	scrub tech	0.03	0.04	0.415	-0.05	0.11
	anesthetist	-0.20	0.04	0.000	-0.28	-0.12
anesthetist	attending	0.12	0.04	0.005	0.04	0.20
	resident	0.25	0.04	0.000	0.17	0.33
	student	0.18	0.05	0.001	0.07	0.29
	scrub tech	0.23	0.04	0.000	0.16	0.31
	circulator	0.20	0.04	0.000	0.12	0.28

Phase 2: First third of the operation

		Mean Difference (I-J)	SE	Sig.	95% Confidence Interval	
					Lower Bound	Upper Bound
attending	resident	0.01	0.05	0.911	-0.10	0.11
	student	0.14	0.07	0.045	0.00	0.28
	scrub tech	0.26	0.05	0.000	0.16	0.36
	circulator	0.16	0.05	0.003	0.06	0.26
	anesthetist	0.29	0.05	0.000	0.19	0.40
resident	attending	-0.01	0.05	0.911	-0.11	0.10
	student	0.13	0.07	0.053	0.00	0.27
	scrub tech	0.25	0.05	0.000	0.15	0.36
	circulator	0.15	0.05	0.003	0.05	0.25
	anesthetist	0.29	0.05	0.000	0.18	0.39
student	attending	-0.14	0.07	0.045	-0.28	0.00
	resident	-0.13	0.07	0.053	-0.27	0.00
	scrub tech	0.12	0.07	0.080	-0.01	0.25
	circulator	0.02	0.07	0.782	-0.11	0.15
	anesthetist	0.15	0.07	0.027	0.02	0.29
scrub tech	attending	-0.26	0.05	0.000	-0.36	-0.16
	resident	-0.25	0.05	0.000	-0.36	-0.15
	student	-0.12	0.07	0.080	-0.25	0.01
	circulator	-0.10	0.05	0.046	-0.20	0.00
	anesthetist	0.03	0.05	0.529	-0.07	0.13
circulator	attending	-0.16	0.05	0.003	-0.26	-0.06
	resident	-0.15	0.05	0.003	-0.25	-0.05
	student	-0.02	0.07	0.782	-0.15	0.11
	scrub tech	0.10	0.05	0.046	0.00	0.20
	anesthetist	0.13	0.05	0.010	0.03	0.23
anesthetist	attending	-0.29	0.05	0.000	-0.40	-0.19
	resident	-0.29	0.05	0.000	-0.39	-0.18
	student	-0.15	0.07	0.027	-0.29	-0.02
	scrub tech	-0.03	0.05	0.529	-0.13	0.07
	circulator	-0.13	0.05	0.010	-0.23	-0.03

Additional file 5

Phase 3: Middle third of the operation

		Mean Difference (I-J)	SE	Sig.	95% Confidence Interval Lower Bound	Upper Bound
attending	resident	-0.03	0.06	0.567	-0.15	0.08
	student	0.05	0.08	0.487	-0.10	0.20
	scrub tech	0.25	0.06	0.000	0.13	0.36
	circulator	0.22	0.06	0.000	0.11	0.33
	anesthetist	0.22	0.06	0.000	0.10	0.33
resident	attending	0.03	0.06	0.567	-0.08	0.15
	student	0.09	0.08	0.249	-0.06	0.24
	scrub tech	0.28	0.06	0.000	0.17	0.40
	circulator	0.25	0.06	0.000	0.14	0.37
	anesthetist	0.25	0.06	0.000	0.13	0.36
student	attending	-0.05	0.08	0.487	-0.20	0.10
	resident	-0.09	0.08	0.249	-0.24	0.06
	scrub tech	0.20	0.07	0.009	0.05	0.34
	circulator	0.17	0.07	0.025	0.02	0.31
	anesthetist	0.16	0.08	0.032	0.01	0.31
scrub tech	attending	-0.25	0.06	0.000	-0.36	-0.13
	resident	-0.28	0.06	0.000	-0.40	-0.17
	student	-0.20	0.07	0.009	-0.34	-0.05
	circulator	-0.03	0.06	0.601	-0.14	0.08
	anesthetist	-0.03	0.06	0.554	-0.15	0.08
circulator	attending	-0.22	0.06	0.000	-0.33	-0.11
	resident	-0.25	0.06	0.000	-0.37	-0.14
	student	-0.17	0.07	0.025	-0.31	-0.02
	scrub tech	0.03	0.06	0.601	-0.08	0.14
	anesthetist	0.00	0.06	0.931	-0.12	0.11
anesthetist	attending	-0.22	0.06	0.000	-0.33	-0.10
	resident	-0.25	0.06	0.000	-0.36	-0.13
	student	-0.16	0.08	0.032	-0.31	-0.01
	scrub tech	0.03	0.06	0.554	-0.08	0.15
	circulator	0.00	0.06	0.931	-0.11	0.12

Additional file 5

Phase 4: Last third of the operation

		Mean Difference (I-J)	SE	Sig.	95% Confidence Interval Lower Bound	Upper Bound
attending	resident	-0.03	0.04	0.388	-0.10	0.04
	student	-0.09	0.05	0.047	-0.18	0.00
	scrub tech	0.04	0.03	0.262	-0.03	0.11
	circulator	-0.02	0.03	0.501	-0.09	0.04
	anesthetist	-0.07	0.04	0.061	-0.14	0.00
resident	attending	0.03	0.04	0.388	-0.04	0.10
	student	-0.06	0.05	0.189	-0.15	0.03
	scrub tech	0.07	0.03	0.042	0.00	0.14
	circulator	0.01	0.03	0.819	-0.06	0.07
	anesthetist	-0.04	0.03	0.311	-0.10	0.03
student	attending	0.09	0.05	0.047	0.00	0.18
	resident	0.06	0.05	0.189	-0.03	0.15
	scrub tech	0.13	0.04	0.004	0.04	0.22
	circulator	0.07	0.04	0.127	-0.02	0.15
	anesthetist	0.02	0.04	0.592	-0.06	0.11
scrub tech	attending	-0.04	0.03	0.262	-0.11	0.03
	resident	-0.07	0.03	0.042	-0.14	0.00
	student	-0.13	0.04	0.004	-0.22	-0.04
	circulator	-0.06	0.03	0.059	-0.13	0.00
	anesthetist	-0.10	0.03	0.002	-0.17	-0.04
circulator	attending	0.02	0.03	0.501	-0.04	0.09
	resident	-0.01	0.03	0.819	-0.07	0.06
	student	-0.07	0.04	0.127	-0.15	0.02
	scrub tech	0.06	0.03	0.059	0.00	0.13
	anesthetist	-0.04	0.03	0.197	-0.11	0.02
anesthetist	attending	0.07	0.04	0.061	0.00	0.14
	resident	0.04	0.03	0.311	-0.03	0.10
	student	-0.02	0.04	0.592	-0.11	0.06
	scrub tech	0.10	0.03	0.002	0.04	0.17
	circulator	0.04	0.03	0.197	-0.02	0.11
